# Delays in third-line antiretroviral therapy and outcomes in North West province

**DOI:** 10.4102/sajhivmed.v23i1.1394

**Published:** 2022-10-24

**Authors:** Babalwa Majova, Ebrahim Variava, Neil Martinson

**Affiliations:** 1 Department of Internal Medicine, Faculty of Health Science, University of the Witwatersrand, Johannesburg, South Africa; 2 Department of Internal Medicine, Tshepong Hospital, Department of Health North West Province, Klerksdorp, South Africa; 3 Perinatal HIV Research Unit (PHRU), University of the Witwatersrand, Johannesburg, South Africa; 4 Johns Hopkins University Center for TB Research, Baltimore, United States of America

**Keywords:** HIV, third-line antiretroviral therapy, viral resistance, delays

## Abstract

**Background:**

Rapid switching from second-line to third-line antiretroviral therapy (TLART) is crucial for achieving viral suppression and reducing illness related to ART failure.

**Objectives:**

This retrospective cohort study quantified the waiting periods for TLART initiation after virological failure on second-line therapy was detected, assessed factors associated with delays and assessed the outcomes of patients started on TLART.

**Method:**

Data were abstracted from records of individuals eligible for TLART, and the time to TLART initiation was calculated. Reasons for delays were categorised according to patient, clinician and administrative processes.

**Results:**

Fifty-four patients were eligible for TLART. The median delay from the date of first viral load > 1000 copies/mL on second-line therapy to the start of TLART was 640 days (interquartile range [IQR]: 451–983 days). Of the patients that failed second-line and had an application for TLART, 41 (75.6%) were eventually initiated on TLART, and 11 (20.4%) died while waiting. Delays were primarily due to non-response to the first unsuppressed viral load while on second-line ART: 467 days (IQR: 232–803 days).

**Conclusion:**

This study showed a prolonged waiting period for TLART initiation mainly between detected high viral load to requesting of resistance tests; many factors could have contributed, including clinicians’ delayed responses to elevated viral loads. Mortality was high before TLART could be initiated. The process of TLART initiation needs to be made more efficient. Healthcare services should be strengthened to (1) recognise and manage virological failure early and identify those eligible for resistance testing, (2) ensure access to resistance testing and appropriately skilled clinicians, and (3) streamline approvals and delivery of TLART.

## Background

South Africa has the largest HIV epidemic and the largest population on antiretroviral therapy (ART) globally. In 2018, it was estimated that 62% of approximately 7.5 million HIV-infected individuals were on ART.^1^ Treatment with ART is continuously evolving as international and local guidelines change.^1^ South Africa’s first guidelines restricted ART to those with CD4 < 200 cells/mm^3^ and those with World Health Organization (WHO) stage 3 or 4 disease.^2^ Since then, ART eligibility criteria have evolved, and in 2016, a Test and Treat policy^3^ was implemented, recommending ART initiation for any person testing positive for HIV, regardless of CD4 count or WHO stage.^3,4^ Despite these advances, HIV drug resistance remains a serious obstacle to virological suppression and good health outcomes. Resistance may be due to a complex interaction of multiple factors, including poor adherence, drug interactions, the low genetic barrier to resistance of some antiretroviral medications, drug stock-outs and delays in regimen switching after virological failure.^5,6^

At the time of this study, patients who failed non-nucleoside reverse transcriptase inhibitor (NNRTI) based first-line regimens were routinely switched to protease inhibitor (PI)-based second line.^7^ Second-line failure was defined as two viral loads > 1000 copies/mL six months apart.^4^ During the period relevant to this study, the criteria for third-line eligibility were: > 1 year of a PI-based regimen with virological failure despite adherence support and evidence of PI resistance.^8,9,10^ During the period of this study, clinicians (doctors and nurse practitioners) could diagnose virological failure if a patient had been on a PI-based regimen for more than a year^9^ and had two detectable viral loads taken six months apart. Only then was a viral resistance test requested. Only when resistance mutations compromising lopinavir/ritonavir or atazanavir/ritonavir (the two PIs available for second-line) were present could a third-line regimen be considered.^9,10^

The protocol of switching patients to third-line antiretroviral therapy (TLART) in South Africa is a complex process requiring diagnosis, approval by a committee of experts, procurement of medication, delivery of medications, monitoring adherence and outcomes. An application is made to the TLART Committee, and only once their approval has been obtained and appropriate regimens recommended can provincial and hospital pharmacies make arrangements for procurement of the proposed regimen. Delays may occur at various points during this process, from recognition of virological failure to requesting resistance tests, obtaining results, submitting TLART applications, and receiving replies and recommendations from the TLART Committee. For this study, these delays have been classified as patient-related, clinician-related or administrative delays. The aim of this study was to quantify delays in TLART initiation, identify the consequences for patients and investigate factors associated with delays.

## Methods

### Design

This retrospective cohort study examined the clinical records of adult patients receiving second-line ART with documented PI resistance. The review period was between June 2015 and June 2019 in three hospitals in North West province, South Africa. This is the period when 2015 HIV guidelines were in place. This study was conducted in three district hospitals each serving a wide range of communities in the province where some patients travel long distances to hospital and some use government transport from local clinics and others use their own transport. In these hospitals second-line patients are mainly managed by doctors assisted by nursing staff. Two of these hospitals (Klerksdorp/Tshepong hospital complex and Potchefstroom hospital) have specialist support, while Joe Morolong (previously known as Vryburg) hospital gets telephonic specialist support from Tshepong hospital.

### Data collection

Data were extracted from three sources: hospital TLART databases, the National Health Laboratory Service (NHLS), and Tier.Net, a national ART patient medical record and database. The resulting database has information on viral load tests, including the collection dates and results at the time of the initial diagnosis of second-line failure, HIV resistance assay results showing PI resistance and the date each specimen was taken, the dates the first application form was completed and then submitted to the TLART Committee, the date of response from the TLART Committee with ART recommendations and, finally, the date TLART was initiated.

Further data were retrieved from individual hospital patient records to characterise factors contributing to delays. These included the date the doctor reviewed the patient’s PI resistance results, the date of the first-ever TLART prescription and the date it was dispensed, and the date patients returned to initiate TLART. We also documented the following adverse events occurring before TLART initiation: hospitalisation, opportunistic infections (WHO stage 3 and 4 infections), and death.

### Data analysis

Data were analysed using Stata 14.2 with a significance threshold of *P* < 0.05. Key variables were average overall delay for TLART initiation and patient, clinician, and administration-related factors associated with the delay.

#### Delays in third-line antiretroviral treatment initiation

For this study, the overall delay was defined as the time from the first detected viral load (≥ 1000 copies/mL) after completing at least a year on second-line ART to the date the patient received TLART. The overall delay was stratified by hospital, age category and gender. The rank-sum test and the K-test for equality of medians were used to explore differences in total delay for the different strata.

#### Effects of the delay on patients

The proportion and the characteristics of those who died while waiting for TLART, those who were suppressed on TLART and factors related to suppression are also reported. The scores obtained from the laboratory database were defined as the sum of each mutation penalty score for a drug. Stanford scores were used to categorise resistance: less than 10 indicated susceptible; scores between 10 and 14 indicated potential low-level resistance; scores between 15 and 29 indicated low-level resistance; scores between 30 and 59 indicated intermediate resistance; scores of 60 or greater indicated high-level resistance.

#### Clinician-related delays

Clinician-related delays for the purpose of this study were: reaction to initial elevated viral loads in patients who have been on a second-line ART regimen for more than one year, delays in resistance testing once criteria for testing are met, and delay in drafting the application to the TLART Committee. The median time from the first viral load result > 1000 copies/mL after at least a year on second-line ART to the date of application to the TLART Committee was determined and then stratified by age, gender, hospital and number of elevated viral loads while on second-line treatment.

#### Administration-related delays

Administrative delays evaluated, first, the time for responses from the TLART Committee, calculated from original submission to TLART Committee until the doctor received approval. Second, pharmacy-related delays considered the availability of TLART medications on the day of the first prescription to identify if drug availability contributed to TLART initiation delays. Administrative delays were stratified by age, gender and by study hospital.

#### Patient-related delays

For this study, patient-related factors were defined as missed appointment dates or failing to collect treatment on the prescription date. A univariate and multivariable binomial model was used to determine patient-level factors independently associated with waiting times > 6 months. Patient factors included in multivariable analyses were age, gender, clinical history and viral loads. Variables with *P* < 0.25 in univariate analyses were included in the multivariable model.

### Ethical considerations

Approval was obtained from the University of Human Research Ethics Committee (reference number: R14/49, protocol number: M200918) and from the chief executive officers and the heads of internal medicine departments at the three study hospitals.

## Results

**FIGURE 1:**
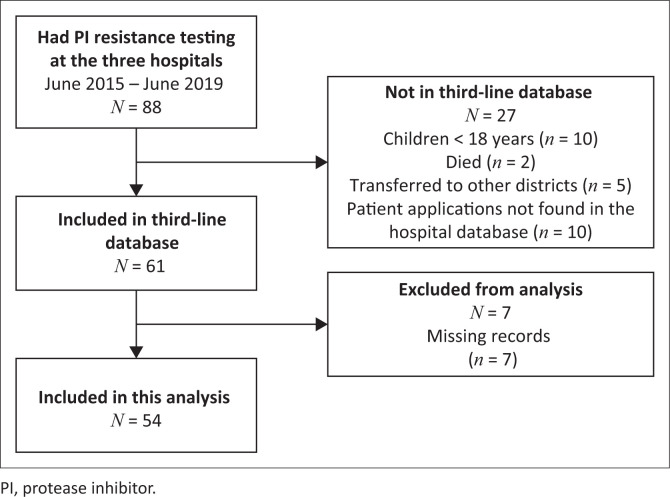
Study schema of patients diagnosed with protease inhibitor resistance on second-line antiretroviral treatment and identified as being candidates for third-line antiretroviral treatment in North West, South Africa.

During the study period 88 patients with PI resistance testing were registered at the NHLS in North West and 54 patients were enrolled into the study. Reasons for exclusion are shown in Figure 1.

### Description of patients who failed second-line therapy

The baseline characteristics and outcomes of the 54 study patients are shown in Table 1. The median duration on ART from first-ever ART initiation to PI resistance being diagnosed was 5.5 years (interquartile range [IQR]: 6–11 years).

**TABLE 1:** Baseline characteristics and outcomes of individuals who failed second-line therapy with protease inhibitor resistance in North West, South Africa, 2015–2019.

Variable	*n*	%	Median interquartile range
**Age (years)**	45.5	-	39–50
**Birth gender**			
Female	32	59.3	-
Male	22	40.7	-
**Hospital**			
Potchefstroom	12	22.2	-
Tshepong	37	68.5	-
Vryburg	5	9.3	-
**Number of viral load measurements during second-line after detecting > 1000 copies/mL**	3	-	3–3
**Hospitalised while waiting for TLART**	16	29.6	-
**Diagnosed with an opportunistic infection while waiting for TLART**	24	44.4	-
**Died while waiting for TLART**	11	20.4	-

TLART, third-line antiretroviral therapy.

### Time from first unsuppressed viral load to third-line antiretroviral treatment application

**FIGURE 2:**
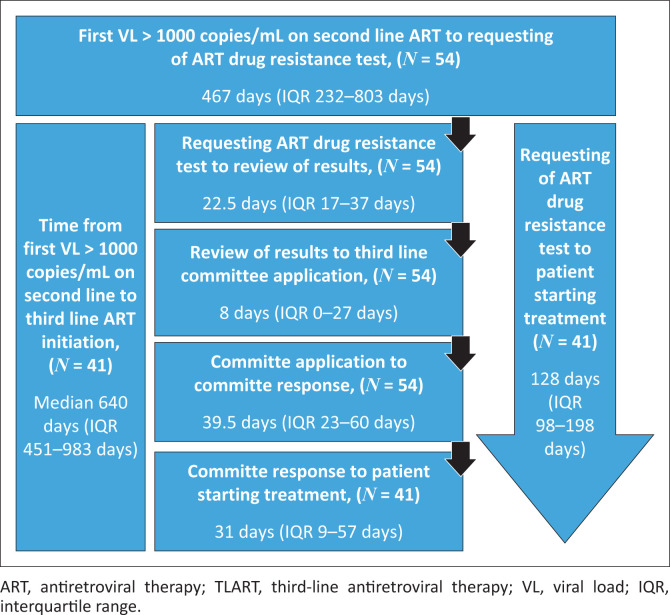
Overall delay to initiation of third-line antiretroviral therapy (TLART) and stratification of delays showing median time (days) from the date the first viral load > 1000 copies/mL on second-line therapy to the date that TLART was started.

The median delay from first viral load > 1000 copies/mL of the two viral loads done 6 months apart to submission of application to the TLART committee in the 54 study patients was 500 days (IQR: 301–805 days) (Figure 2) and 36 (66.7%) were longer than one year. Most of the delays were in the period between detection of VL > 1000 copies/mL to requesting ART drug resistance test. Most of these patients had three viral load assays collected after first detecting > 1000 copies/mL before a specimen for HIV resistance testing was taken. The time from requesting ART resistance test to reviewing of results was 22.7 days (IQR: 17–37 days). The time from the clinicians receiving the resistance test results and submission of the TLART Committee was 8 days (IQR: 0–27 days). The overall period from requesting ART drug resistance test to starting TLART was 128 days (IQR: 98–198 days) (Figure 2).

### Analyses of variables associated with time from first viral load > 1000 copies/mL to Committee application

Analyses of categorical variables associated with time to TLART application are shown in Table 2. The total waiting time in days was analysed looking at the patient characteristics and the number of viral loads done during that period was statistically significant.

**TABLE 2:** Analyses of variables associated with first viral load > 1000 copies/mL to the Committee application being submitted.

Characteristics	Total waiting time in days		*P*-value for rank-sum test
	Median	Interquartile range	
**Age (*N* = 54)**			
≥ 40 years	499	301–908	-
< 40 years	500	259–717	0.885
**Birth gender**			
Women	500	307–739	-
Men	517	197–908	0.972
**Developed opportunistic infections in waiting period**			
No	454	313–750	-
Yes	598	178–871	0.747
**Hospitalised during the waiting period**			
No	500	313–911	-
Yes	524	178–704	0.501
**Year of ART start**			
2009 or earlier	565	328–935	-
2010 or later	405	197–753	0.205
**Year of second-line start**			
2013 or earlier	500	219–935	-
2014 or later	494	313–750	0.951
**Number of VLs > 1000 copies/mL on second-line counted after the first VL > 1000**			
≥ 3	534	333–961	-
< 3	255	109–649	0.018
Potchefstroom hospital	650	442–1136	-
Tshepong hospital	399	206–750	-
Vryburg hospital	517	499–620	0.331*

ART, antiretroviral therapy; VL, viral load.

* , K-test for equality of medians.

### Viral suppression in those initiated on third-line treatment

A total of 41 patients were started on TLART and 32 (74.4%) had viral load suppression 3–12 months after initiation of TLART. Some of these patients developed opportunistic infections during the time when they were still waiting for TLART. They had adverse events while waiting to initiate TLART, either an opportunistic infection or hospitalisation during the waiting period.

## Discussion

This retrospective cohort study describes people living with HIV who failed second-line therapy and survived to have an application submitted to the South African TLART Committee. The median delay to initiate TLART was almost 18 months while patients continued ineffective second-line therapy. The longest delays occurred in recognising and diagnosing second-line treatment failure by clinicians. The consequences of delaying a switch to more effective ART include accumulation of resistance mutations, opportunistic infections, hospitalisations and death. Indeed, 20% of the cohort died in this study while waiting for TLART. Similar negative consequences of delayed switching are described in Tanzania, which showed that delayed switching increased the risk of opportunistic infections and Shroufi et al. (2019) estimated that avoiding delayed switching can prevent 10 215 deaths annually in South Africa.^9,11^ The adverse outcomes described in this study could be reduced if patients with treatment failure were identified earlier and timeously switched to a more effective regimen. This study shows that approximately three-quarters of patients who switched to third-line therapy achieved viral suppression.

The delays reported in this study are similar to those observed in two South African studies that examined delays in switching to second-line regimens,^6,9,11^ as well one of the few studies that showed significant delays in switching patients to second-line ART.^13^ While there is no recommended time the process should take, there is global consensus that earlier switching of ART improves patient outcomes for those on failing regimens.^4,5,12,13,14,15,16,17,18^

This study identified factors associated with switching delays, including clinician-related and administrative factors such as delayed response from the national TLART Committee and delayed applications for switching. There are no studies that have examined these delay factors. Others have reported modifiable programmatic factors such as insufficient prescriber notes and lack of availability of viral load results identified with delays in switching to second-line regimens in a study conducted in Durban, South Africa.^13^ A study from the Western Cape found that factors associated with the delays were predominantly health system-related and mainly pertained to patient referrals.^17^

While this study provides helpful insight into the process of TLART initiation, it was subject to some limitations. First, this study did not investigate the clinician’s knowledge of the proper process for TLART application nor their knowledge of advanced HIV management. Second, processes of the TLART Committee were not fully examined. Third, findings are likely to be context specific, and will be influenced by regional resource limitations such as staff capacity, training levels, efficiency of referral pathways, lab capacity and other health system factors. Finally, this study was limited to patients with identified PI resistance – a full understanding of the cascade of care for TLART should include all patients experiencing virologic failure on second-line ART.

Future studies should assess the entire process the TLART Committee follows to make the final decision and evaluate what influences clinicians’ responses to high HIV viral loads and PI resistance results.

## Conclusion

We found prolonged waiting periods between virological failure and resistance testing, and between PI resistance detection and the initiation of TLART. Severe adverse events (opportunistic infections, hospitalisation and death) occurred during these delays. The processes ending in TLART initiation needs to be made more efficient. Healthcare services should be strengthened to (1) recognise and manage virological failure early and identify those eligible for resistance testing, (2) ensure access to resistance testing and appropriately skilled clinicians, and (3) streamline approvals and delivery of TLART. The results compel further exploration into the many factors affecting these process steps to identify specific areas for quality improvement.
